# The prognostic value of the head-shaft angle on hip displacement in children with cerebral palsy

**DOI:** 10.1007/s11832-015-0654-z

**Published:** 2015-04-29

**Authors:** J. P. J. van der List, M. M. Witbreuk, A. I. Buizer, J. A. van der Sluijs

**Affiliations:** Department of Orthopaedic Surgery, Research Institute MOVE, VU University Medical Center, De Boelelaan 1117, P.O. Box 7057, 1081 HV Amsterdam, The Netherlands; Department of Rehabilitation Medicine, VU University Medical Center, De Boelelaan 1117, P.O. Box 7057, 1081 HV Amsterdam, The Netherlands

**Keywords:** Head-shaft angle, HSA, Hip displacement, Hip subluxation, Cerebral palsy

## Abstract

**Background:**

Hip displacement is the second most common deformity in cerebral palsy (CP). The risk for hip displacement is related to the Gross Motor Function Classification System (GMFCS). Recently, the head-shaft angle (HSA) has been identified as a predictor for hip displacement and the aim of this study is to assess the predictive value of the HSA for hip displacement in CP.

**Methods:**

In this retrospective cohort, we performed radiological measurements in 50 children on both hips. In children with GMFCS level II (30 hips), III (30 hips), IV (20 hips) and V (20 hips), we measured the HSA and migration percentage (MP) in three age intervals: age two years (T1), age four years (T2) and age seven years (T3).

**Results:**

At T1, the HSA was larger (more valgus) in hips that will displace than in hips that will not displace (174° vs. 166°; *p* = 0.001) and was also larger in higher GMFCS levels (IV–V vs. II–III) (172° vs. 165°; *p* < 0.001). At T1, GMFCS [odds ratio (OR) 14.7; *p* = 0.001] and HSA (OR 1.102; *p* = 0.043) were predictors for hip displacement at T3, but at T2, MP (OR 1.071; *p* = 0.010) was the only predictor for hip displacement at T3.

**Conclusions:**

The HSA at two years is larger in hips that will displace and larger in children with higher GMFCS levels (IV–V). At age two years, GMFCS and HSA are valuable predictors for hip displacement, but at the age of four years, only MP should be used in the prediction of hip displacement.

**Level of evidence:**

Prognostic study, level II.

## Introduction

In Europe, cerebral palsy (CP) is the most common cause of physical disability in childhood, with an incidence of 2 in 1000 live births [1]. CP is a static encephalopathy that results in a high muscle tone, paresis, muscle contractures, joint instability and musculoskeletal deformities, resulting in activity limitations. After equinus, hip displacement is the second most common deformity in CP [2]. The most important consequences of hip displacement are pain, degenerative osteoarthritis, windblown hip syndrome and problems with sitting, standing and walking [3, 4]. To prevent these symptoms, studies point out that soft tissue release, performed before the age of five years, is indicated when a hip has a Reimers’ migration percentage (MP) above a threshold of 40 % [3, 5] or when the yearly increase of MP exceeds 10 % [3]. For prevention of hip displacement in CP, children screening programmes are used. The age for initial radiography for screening varies between several studies, but should generally be performed before the age of 30 months or earlier if abnormal clinical signs are present [6, 7]. Larnert et al. [8] stated that the annual incidence for hip displacement is highest between two and three years of age and decreases with each year of age until seven years of age, when a plateau is reached. The risk of hip displacement is related to the level of mobility of children with CP as classified by the Gross Motor Function Classification System (GMFCS) [9], with a prevalence of 0 % in GMFCS I to 64 % in GMFCS V [5]. Children with GMFCS I and II are able to walk and climb stairs without assistive devices, children with GMFCS III are able to walk with assistive mobility devices, while children with GMFCS IV and V are mainly dependent on a wheelchair for their mobility [9]. Another study supports that the yearly increase of MP is dependent on the GMFCS and is a risk factor for hip displacement [10]. Thus, the radiological follow-up scheme should depend on the initial MP, the yearly increase of MP and the GMFCS [7, 11].

Besides these parameters, a recent study showed that neck-shaft angle (NSA) and head-shaft angle (HSA) have a correlation with MP and could be useful parameters for the prediction of hip displacement [12]. The HSA has advantages over the NSA, since its intra- and interrater reliability is good and the HSA is less influenced by rotation of the femur compared to the NSA [13]. Not only is the HSA related to MP at seven years of age, but at young age, it also seems to be a useful predictor for hip displacement (MP ≥40 %). The HSA at two years of age was a predictor for hip displacement at a later age, independent of GMFCS and MP at two years of age [14].

The aim of this study is to assess the predictive value of HSA in the first years of life for subsequent hip displacement in CP. The hypothesis of this study is that the growth and orientation of the proximal femoral epiphysis is influenced by muscle imbalance in CP with over-activity of adductors and iliopsoas versus weakness of abductors [15, 16], resulting in a more horizontal orientation of the femoral epiphysis and, thus, a larger HSA in the CP hips [16]. This altered orientation means that the hip develops in a valgus direction [15] and it is likely that coxa valga plays a role in hip displacement [17]. So, we expect a relation between a larger HSA at young age and a higher incidence of hip displacement (MP ≥40 %) at seven years of age, and we expect this relationship to be stronger at the higher GMFCS levels.

## Patients and methods

### Patients

We conducted a retrospective cohort study in which we selected children from a database of CP children referred to our institute between January 1, 2001 and August 1, 2009. The inclusion criteria were: spastic bilateral CP, GMFCS level II or higher [7] and availability of anteroposterior (AP) radiograph of the pelvis in three time intervals: age of two years (12–32 months; T1), age of four years (36–60 months; T2) and age of seven years (72–96 months; T3). We excluded five patients who underwent dorsal rhizotomy or varus osteotomy before the radiograph at T3. Five patients were included even though they underwent unilateral soft tissue release within six months before the radiograph at T3. These five hips were displaced at T2 and were still displaced at T3. Further details are provided in the discussion. We did not select patients with GMFCS I because radiological follow-up is not indicated [7] and hip displacement in these patients is rare [5]. All patients received physiotherapy as a part of standard care.

After reviewing 535 children from the database on the inclusion and the exclusion criteria, 50 children met the criteria: 15 patients with GMFCS level II, 15 patients with GMFCS level III, 10 patients with GMFCS level IV and 10 patients with GMFCS level V. Parameters assessed at age of two years were GMFCS level and gender, and their GMFCS classification was adjusted through the years of follow-up, if necessary. We performed measurements on both hips, so we included a total of 30 hips with GMFCS level II, 30 hips with GMFCS level III, 20 hips with GMFCS level IV and 20 hips with GMFCS level V.

### Radiographic measurements

**Fig. 1:**
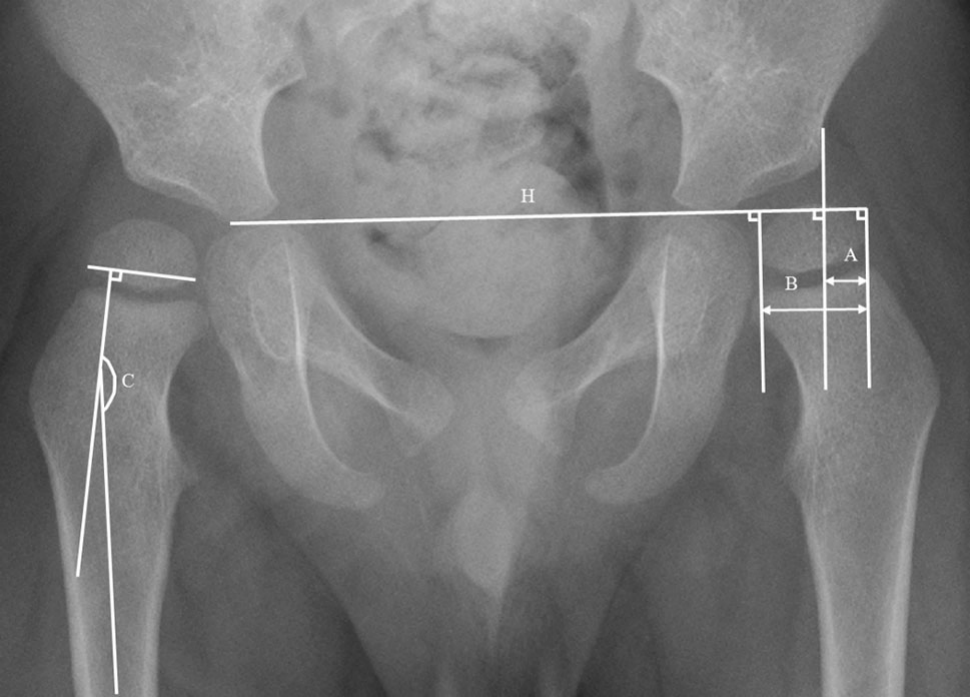
Anteroposterior (AP) radiograph of the pelvis. Right hip: head-shaft angle (*C*) by measuring the medial angle between a line perpendicular to the proximal femoral epiphysis and a line through the middle of the femoral shaft. Left hip: the migration percentage (MP) is measured by a Hilgenreiner’s line (*H*) and three perpendicular lines. The MP is measured by *A*/*B* × 100 %

The two radiographic parameters, HSA and MP, were assessed at T1 (between 12 and 32 months), at T2 (between 36 and 60 months) and at T3 (between 72 and 96 months) in a total of 100 hips. We used the AP radiograph of pelvic and hip joints to measure the AP HSA and MP at these ages. Standardised radiographs were obtained with the patient supine, the pelvis symmetrically positioned with the spinae iliacae on equal height and straightened legs with 20° internal rotation. The HSA is measured on an AP radiograph according to Southwick [18] (Fig. 1) and his measurement has a maximal intrarater variability of 1.7 % and a maximal interrater variability of 1.8 % at the AP radiograph [19]. The MP, as described by Reimers [20], was used to measure the lateral displacement of the femoral head (Fig. 1). The MP is not influenced by rotation of the hip [20] and has an intrarater variability of 3.6 % and an interrater variability of 3.2 % [21, 22]. Because of the low intra- and interrater variability of both the HSA and the MP, the measurements were performed by the same examiner (JL) once. They were performed on the digital radiology system IMS Webviewer [23].

### Statistical analysis

For the statistical analysis, we divided the patients into GMFCS levels II–III and GMFCS levels IV–V, because these groups differ clinically: children in GMFCS levels II–III will be able to walk versus GMFCS levels IV–V, who will not be able to walk [9]. The MP at seven years was classified into two groups: a group without hip displacement (MP <40 %) and a group with hip displacement (MP ≥40 %). This cut-off is chosen because Hägglund et al. [24] found that surgical intervention is indicated when the hip is displaced above this threshold because of expected further dislocation.

All statistical analyses were performed with IBM SPSS Statistics 20 [25]. Independent *t*-tests were used to assess differences in the HSA at different ages between GMFCS levels II–III versus GMFCS levels IV–V and between groups with hips who, at T3, will and will not displace. Correlation analysis was performed to find a correlation between HSA at T1, GMFCS and MP at different ages with a Pearson correlation test. Logistic regression was performed to assess the odds ratio (OR) of GMFCS level, HSA at T1, MP at T1 and age at T3 for hip displacement (MP ≥40 %). We performed the same logistic regression with parameters (HSA and MP) at T2 as independent parameters. In the logistic regression, it is important to correct for the age at T3 because the MP is still developing in this age interval to some extent, so the age could be relevant to the displacement of the hip with the cut-off of MP set at 40 %. A Chi-square test was performed to find the threshold of the HSA in predicting hip displacement.

### Ethics

The study was approved by the Medical Ethical Examination Committee of our institution (no. 2014.523).

## Results

We performed radiological measurements in three different time intervals on 100 hips in 50 children (35 males) with CP. At the end of the follow-up period, 18 out of 100 hips displaced (MP ≥40 %): two hips in GMFCS III; seven hips in GMFCS IV and nine hips in GMFCS V.

**Fig. 2:**
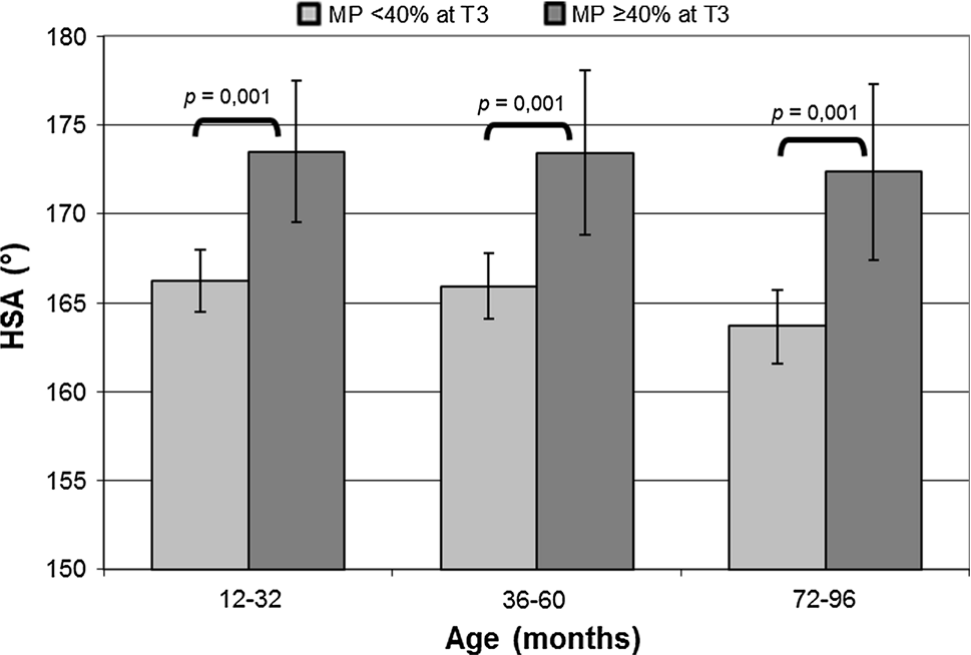
Mean of the head-shaft angle (HSA) in the follow-up intervals of children in hips that eventually displace (MP ≥40 %) or will not displace (MP <40 %) at seven years (T3)

At all ages, the HSA was larger in the hips that displaced (MP ≥40 % at T3) than in hips that did not displace (MP <40 % at T3). At T1, the HSA in hips that will displace was significantly larger [174°; confidence interval (CI) 169°–178°] than the HSA in hips that will not displace (166°; CI 164°–168°; *p* = 0.001). The differences at T2 and T3 were also significant (both *p* = 0.001) (Fig. 2).

**Fig. 3:**
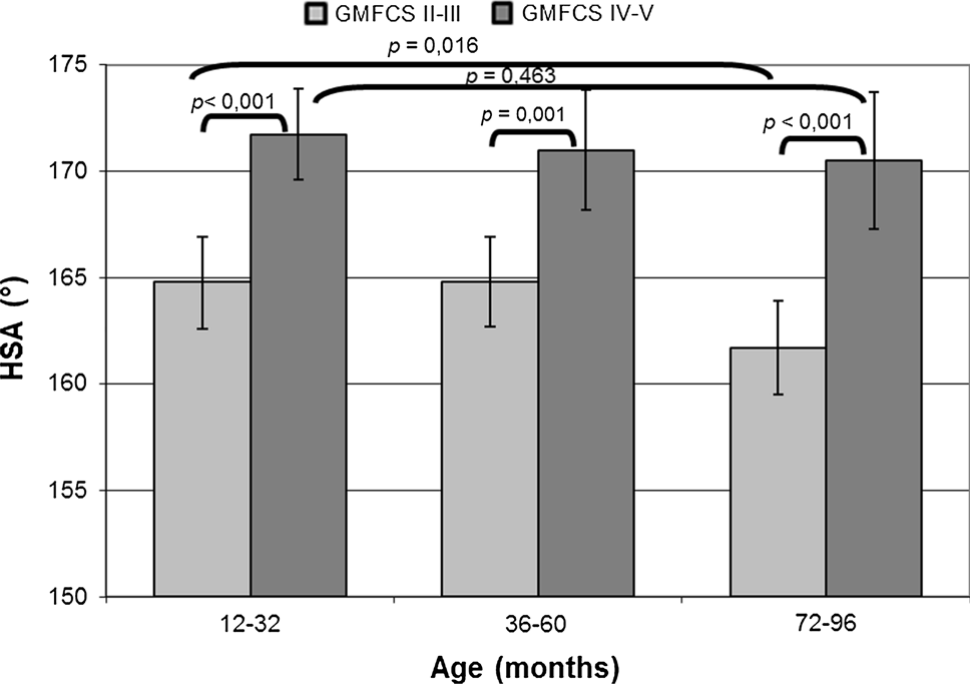
Mean of the head-shaft angle (HSA) in the follow-up intervals of children with Gross Motor Function Classification System (GMFCS) level II–III or IV–V

At T1, the mean HSA in GMFCS levels IV–V was larger (172°; CI 170°–174°) than the HSA in GMFCS levels II–III (165°; CI 163°–167°; *p* < 0.001). This difference was also significant when measured at T2 (171° vs. 164°) and T3 (170° vs. 162°) (Fig. 3).

Correlation analysis showed that GMFCS (0.581; *p* < 0.001), HSA at T1 (0.354; *p* = 0.001) and MP at T1 (0.198; *p* = 0.049) all have a correlation with MP at T3, with GMFCS having the strongest correlation. There is a correlation between GMFCS and HSA at T1 (0.473; *p* < 0.001) as well, but this correlation is not as strong as the correlation between GMFCS and MP at T3 (0.581; *p* < 0.001) (Table 1).

**Table 1:** Correlation between radiographic measurements at different ages

	HSA at T1	MP at T1	MP at T2	MP at T3
HSA at T1		0.112 (*p* = 0.266)	0.278 (*p* = 0.005)	0.354 (*p* = 0.001)
HSA at T2			0.382 (*p* < 0.001)	0.433 (*p* = 0.001)
HSA at T3				0.348 (*p* = 0.001)
GMFCS	0.473 (*p* < 0.001)			0.581 (*p* < 0.001)
MP at T1				0.198 (*p* = 0.049)

*HSA* head-shaft angle, *MP* migration percentage, *T1* radiograph at age 12–32 months, *T2* radiograph at age 36–60 months, *T3* radiograph at age 70–96 months

Logistic regression at T1 was performed to find the prognostic value of the HSA and we used hip displacement (MP ≥40 % at T3) as the dependent parameter. As the independent parameters, we used GMFCS (II–III vs. IV–V), HSA at T1, MP at T1 and age at T3. The analysis showed that both GMFCS (OR 14.7; CI 3–74; *p* = 0.001) and HSA at T1 (OR 1.102; CI 1.006–1.207; *p* = 0.037) are prognostic factors on hip displacement (Table 2). Our model of logistic regression with HSA at T1 and GMFCS had a negative predictive value of 92.7 % and a positive predictive value of 33.3 %.

**Table 2:** Logistic regression for hip displacement (MP ≥40 % at T3) in relation to different prognostic factors

		OR	95 % CI	*p*-Value
GMFCS level	IV–V vs. II–III	14.683	2.917–73.918	0.001
HSA at T1	Degree	1.102	1.006–1.207	0.037
MP at T1	Percentage	0.993	0.948–1.041	0.785
Age at T3	Months	0.948	0.883–1.018	0.144

Nagelkerke *R* ^2^ = 0.410; negative predictive value of 92.7 %; positive predictive value of 33.3 %

*MP* migration percentage, *GMFCS* Gross Motor Function Classification System, *HSA* head-shaft angle, *OR* odds ratio, *CI* confidence interval, *T1* radiograph at age 12–32 months, *T3* radiograph at age 72–96 months

Logistic regression at T2 was performed with hip displacement (MP ≥40 % at T3) as the dependent parameter and GMFCS (II–III vs. IV–V), HSA at T2, MP at T2 and age at T3 as the independent parameters. The analysis showed that at age four years, only MP is relevant as a prognostic value on hip displacement (OR 1.071; CI 1.017–1.128; *p* = 0.010). GMFCS (OR 5.5; *p* = 0.053) and HSA at T2 (OR 1.072; *p* = 0.132) were not significant predictors for hip displacement.

The Chi-square test was performed to find a clinically relevant cut-off value of the HSA. We found that, when the HSA at T1 is lower than 165°, only 3/41 (7 %) of the hips displaced at T3, and when the HSA at T1 is higher than 165°, 15/59 (25 %) of the hips displaced at T3 (*p* = 0.02) (Table 3).

**Table 3:** Cross table with HSA at T1 and hip displacement at T3

HSA at T1	Hip displacement at T3		Total
	≥40 %	<40 %	
≥165°	15	44	59
<165°	3	38	41
Total	18	82	100

Chi-square test *p* = 0.02; sensitivity 83 %; specificity 46 %

*HSA* head-shaft angle, *T1* radiograph at age 12–32 months, *T3* radiograph at age 72–96 months

## Discussion

**Fig. 4:**
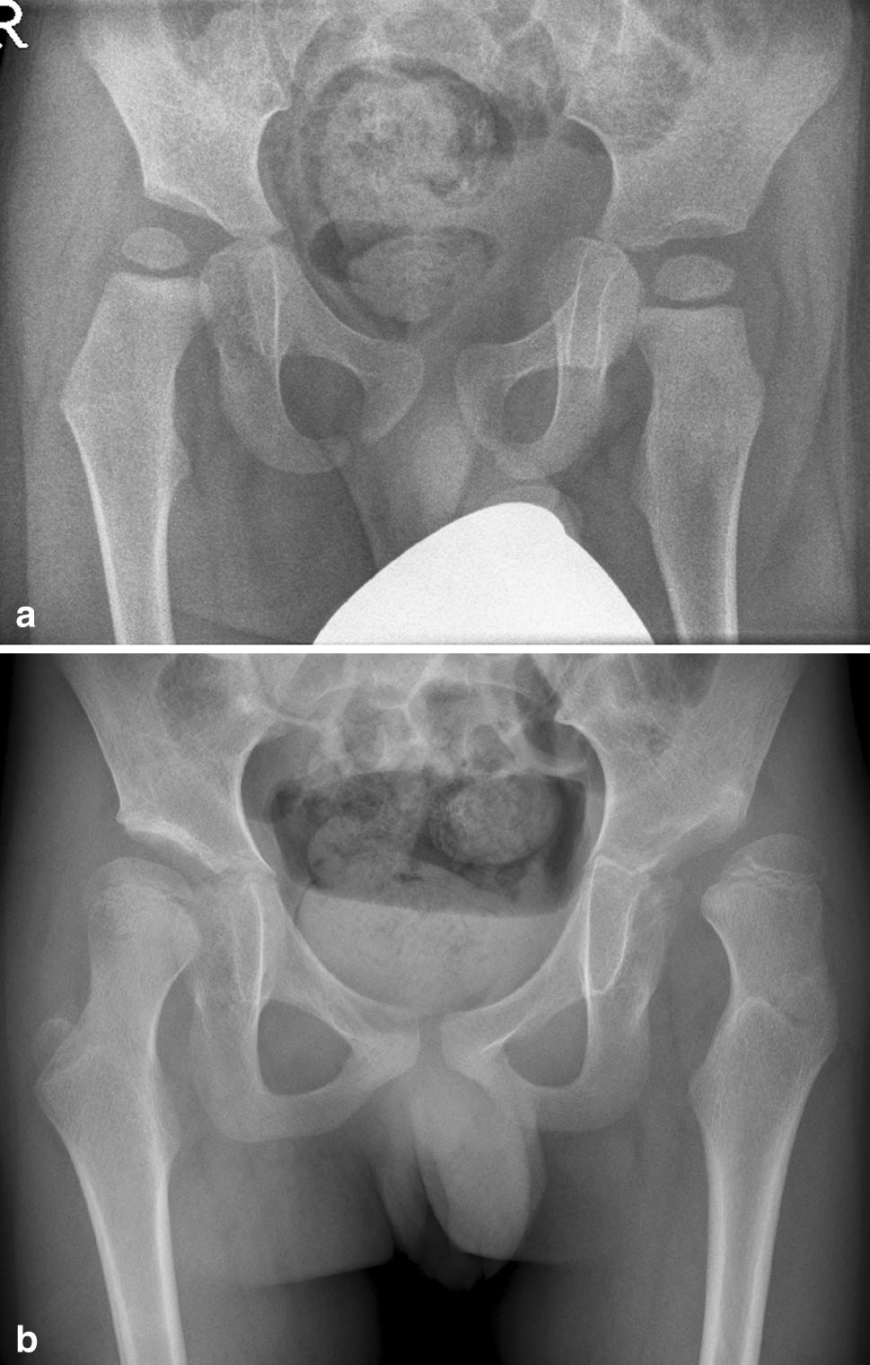
AP radiographs of a boy with GMFCS level V cerebral palsy. **a** At T1 (22 months of age), the patient had a head-shaft angle (HSA) of 169° right and 185° left, with a migration percentage (MP) of 29 % right and 0 % left. **b** At T3 (95 months of age), the right hip was displaced laterally with a MP of 34 % and the left hip with a MP of 76 %

Our study presents retrospective longitudinal data on the HSA in CP children aged two to eight years and confirms the prognostic value of the HSA that has only recently been stressed by Hermanson et al. [14]. The HSA at two years is of prognostic value for hip displacement (OR 1.102) when corrected for GMFCS (II–III vs. IV–V), age at T3 and MP at T1. The HSA and GMFCS have a high negative predictive value in this model. This means that, when two hips are compared at age two years with only a 10° difference in the HSA, the hip with the larger HSA has a three times higher risk of hip displacement than the other hip (1.102 raised to the tenth power) (Fig. 4). In this study, we found a clinically relevant threshold of the HSA of 165°, and this means that the risk of hip displacement at T3 is low when the HSA at age two years (T1) is smaller than 165°, regardless of the GMFCS level. Further studies should assess this threshold value in clinical practice. However, it is important to assess that the HSA at T1 seems to play only a minor role compared to GMFCS. The most important predictor of hip displacement is GMFCS and the HSA plays a small additional role in this prediction. There is a correlation between the HSA at T1 and MP at T3 (0.354) and between GMFCS the and HSA at T1 (0.473), but this latter correlation is not as strong as the correlation between GMFCS and MP at T3 (0.581). It is possible that a part of the prognostic value of the HSA is explained by GMFCS. In this study. GMFCS is the most important prognostic factor for hip displacement and the HSA has an additional prognostic factor to GMFCS.

At age four years (T2), the HSA and GMFCS have no prognostic value for hip displacement at T3 and MP at this age is the only significant predictor for hip displacement at T3 (with an OR of 1.071). Based on the results of this study, the GMFCS level and HSA should be used as prognostic factors for hip displacement in a patient aged two years and the MP should be used as a prognostic factor for hip displacement in a patient aged four years. This finding of MP as a prognostic factor at later age (T2) is consistent with the recent literature [7]. A possible explanation of why GMFCS and HSA are not significant predictors of hip displacement at age four years could be that the hip is on track to hip displacement at this age. This means that at age four years, the MP is a much stronger predictor because an important part of the hip displacement has already taken place (measured by the MP) and this progress of hip displacement is of higher prognostic value than the GMFCS level or HSA at this age (T2).

Although normal reference values of HSA between two and eight years are lacking, it is likely that the HSA decreases through the ages of early childhood in normal children, since the HSA between eight and 16 years of age is smaller [26]. In our study, the HSA decreases in GMFCS levels II and III, but remains unchanged in GMFCS levels IV and V (Fig. 2). We suggest that the muscle imbalance is responsible for this process. Since the orientation of the growth plate is assumed to be perpendicular to the force acting on it [16], a change in the balance between the adductors and abductors would lead to an altered orientation. In CP, the strong adductors and iliopsoas muscle and weak abductor muscles [27] lead to a change in the normal balance that is more severe at the higher GMFCS levels [28]. Because of this muscle imbalance, the forces on the femoral head become more vertical and, as the epiphysis is at right angles to the forces acting on it, the epiphysis becomes more horizontal. Due to this orientation, bone development is influenced and leads to a failure of a normal developing varus in the neck of the femur [15, 16]. This means that, at the higher GMFCS levels, a larger HSA will exist, and this was found in a cross-sectional study [12] and in this longitudinal study. The effect of muscle imbalance on bone growth is also apparent in the abnormal femoral anteversion in CP children: in healthy children, the femoral anteversion decreases in childhood, but in CP children, this anteversion remains stable or is even slightly increased [29], with the femoral anteversion being larger in GMFCS levels IV–V compared to GMFCS levels II–III [30]. The findings of a decrease of femoral anteversion in normal hips and a stronger decrease in GMFCS levels II–III correspond to the findings of the HSA in this study. In CP, it is thought that this larger femoral anteversion and larger HSA, and, thus, a coxa valga, in combination with adduction deformity play a major role in progressive subluxation of the hip [2, 17].

A limitation of this study is the small amount of children in the different GMFCS groups and the small number of displaced hips (*n* = 18). Although both measurements, the HSA and the MP, have high intra-observer reliability, the small number of hips could cause some variability in the results. Another limitation is the treatment in the follow-up period. We excluded patients who underwent dorsal rhizotomy and varus osteotomy and this could be a selection bias. Furthermore, we included patients who underwent a treatment with botulinum toxin type A or received braces, and Graham et al. [31] found that these interventions could have a small treatment benefit, although the effect was not enough to advise treatment with botulinum and braces. Nevertheless, it cannot be excluded that these treatments had a minor effect on the muscle imbalance and, thus, the MP. The five included patients who underwent unilateral soft tissue release had a MP >40 % at T2 and received the release within six months of radiograph at T3. These patients had a MP >40 % at T3 and excluding these patients would be a selection bias to the study because this treatment was given due to the hip displacement. The last limitation of this study is the fact that the HSA was not corrected for the femoral anteversion that could influence the HSA, especially when measured on an AP radiograph that is two-dimensional [2]. However, Foroohar et al. showed that, when the hip on an AP radiograph was 45° rotated, there was only a 5° difference between the measured HSA and the true HSA. Therefore, we think that it is possible to use an AP radiograph to measure the HSA without correcting for the femoral anteversion [13].

Additional studies are needed to assess the exact clinical importance of the HSA and its use in clinical practice. It would also be of value to take a look at the reference values of the HSA in healthy children in early childhood and compare these results to children with CP.

In conclusion, hip displacement in children with CP is a known problem and the HSA is a prognostic factor on hip displacement. In this study, GMFCS (II–III vs. IV–V) and HSA at age two years are predictors for hip displacement at seven years of age, and the MP at age two years is not a prognostic factor. In this study, the prognostic value of the HSA at age two years has a high negative predictive value and is best applicable with a threshold of 165°, although additional studies are necessary to assess the value of the HSA in clinical practice. At age four years, only the MP is a valuable predictor for hip displacement, whereas the HSA and GMFCS are not.
